# Women’s Economic Contribution, Relationship Status and Risky Sexual Behaviours: A Cross-Sectional Analysis from a Microfinance-Plus Programme in Rural South Africa

**DOI:** 10.1007/s10461-021-03566-5

**Published:** 2022-01-21

**Authors:** Janke Tolmay, Louise Knight, Lufuno Muvhango, Tara Polzer-Ngwato, Heidi Stöckl, Meghna Ranganathan

**Affiliations:** 1 Centre for Social Science Research, University of Cape Town, 4.89 Leslie Social Science Building, Rondebosch, Cape Town, South Africa; 2 London School of Hygiene and Tropical Medicine, London, UK; 3 Intervention with Microfinance and Gender Equity, Johannesburg, South Africa; 4 Social Surveys Africa, Johannesburg, South Africa

**Keywords:** HIV/AIDS, Risky sexual behaviour, Microfinance-plus, Women’s empowerment

## Abstract

In sub-Saharan Africa, women bear a disproportionate burden of HIV/AIDS while also facing economic and gender inequalities. To explore the association of women’s economic contribution and relationship status with risky sexual behaviour, this study analysed cross-sectional data from 626 women aged 22 to 84 in rural South Africa. All women were enrolled in a microfinance plus gender training programme (Intervention with Microfinance for AIDS and Gender Equity (IMAGE)). We used univariable and multivariable logistic regression to explore the associations of relationship status and women’s household income contribution with inconsistent condom use, multiple sexual partners and transactional sex, respectively. We found that married, older women had the highest odds of inconsistent condom use, while those contributing all the household income had higher odds of multiple sexual partnerships, but lower odds of transactional sex compared to those with no contribution. Income contribution and relationship status have a nuanced relationship with sexual risk behaviours. Thus, economic strengthening interventions should target relevant vulnerable women while also addressing the broader social and economic drivers of risky sexual behaviour.

## Introduction

Sub-Saharan Africa bears a disproportionate burden of HIV/AIDS; this region is home to 71% of all people living with HIV worldwide [1]. South Africa reports the highest rate of HIV infections, with 7.7 million cases and a general adult (15–49 years) prevalence of nearly 20.4% [2]. Women are disproportionately affected, making up 62.7% of all adults living with HIV in South Africa [2]. Potential reasons for women’s higher risk of HIV infection include biological factors, such as the physiological structure of the female reproductive tract, and socio-economic factors, such as poverty and income inequalities alongside unequal gender norms, that may lead to risky sexual behaviours. The latter may include non-commercial sex in exchange for material goods or money (termed transactional sex) [3, 4], having multiple sexual partners (at least two or more in the past year or concurrently) [5], substance abuse [6], and inconsistent condom use [7, 8]. Gender based violence, including sexual assault and intimate partner violence (IPV), has also been shown to increase HIV infection [9, 10].

### Relationship Status, Socio-Economic Status, and Risky Sexual Behaviours

In sub-Saharan Africa, socio-demographic factors such as younger age, not being in stable or marital partnerships, female-headed households, and early age of first sex have shown to be drivers for risky sexual behaviours in women [11]. Compared to other regions of the world, where marriage or cohabitation is often perceived as a protective factor for sexually transmitted infections (STIs), studies in several sub-Saharan African countries have found that a significant number of new HIV infections [12] and most unprotected sexual acts between sero-discordant couples [13] occurred within marriage. Married women are often unable to negotiate safe sex practices and are afraid of being considered disloyal or distrustful [14, 15]. Divorced or formerly married women are frequently neglected in sexual risk behaviour research but remain at substantial risk, especially regarding multiple sexual partnerships [16]. These risks are often exacerbated by low relationship power and financial dependence, whether within a marital relationship or via casual sexual encounters in return for money or goods [5, 15, 17]. Further, women who experienced IPV or had low relationship power were found to be at higher risk of HIV infection [5]. The exact pathways linking these factors remain unconfirmed, but studies have linked the experience of IPV and controlling partner behaviour with HIV infection through a complex interaction of bidirectional pathways, including through increased risky sex [10, 18–20].

Poverty or economic vulnerability is an important factor contributing to women’s increased risk of engaging in risky sexual behaviours. In Zimbabwe, women’s lower socio-economic status (SES) and food insufficiency was linked to early marriage, mental health problems, and increased reporting of high risk sexual behaviours [21]. In Kenya, low levels of education, lower income, and larger amounts of money and gifts in exchange for sex were all associated with inconsistent condom use [22]. In the patriarchal structure of many Southern African societies, the expectation that the man should be the financial provider in a sexual relationship is common [23]. Women living in poverty often engage in transactional sex for the provision of basic needs, such as food and electricity, but also as a result of immense social pressure to obtain luxury items or lifestyles that they might not otherwise be able to afford [23–25]. Studies have also shown that women have multiple transactional sexual partners, often concurrently, to fulfil different needs or wants, whether it be romantic love, material necessities, or sexual pleasure [17, 26, 27]. While age-concordant transactional relationships are not uncommon, age-disparate sexual relationships between wealthier, older men and poorer young women often carry a larger power imbalance and risk of exploitation, and are associated with lower levels of condom use [23, 28, 29].

### Economic Strengthening Interventions and HIV Risk Behaviours

Women are more likely to be the main breadwinner in female-headed households compared to male-headed households, while they generally earn less than men. They often have less time for market work due to household responsibilities and childcare, and often face additional hardships linked to adolescent pregnancy and family instability [30]. In South Africa, more women are employed in the informal trade sector than men (47.6% vs 30.6%) and they are more likely to perform unpaid work [31], which contributes to gendered poverty.

An approach to alleviating poverty and improving women’s economic situation is through economic strengthening interventions, such as microfinance programmes, that provide small, group-based loans to women to support entrepreneurship and income generation [32–34]. When microfinance is coupled with gender-focused complementary programmes (microfinance-plus), it has shown positive effects on addressing both HIV risk behaviours and gender inequalities by helping women acquire new business skills that may increase their self-confidence and social capital, help them build support networks, and increase their relationship decision-making power [35, 36]. However, the effectiveness of microfinance-plus programmes vary depending on programme design, duration, target group, and outcomes measured [36–38].

The Intervention with Microfinance for AIDS and Gender Equity (IMAGE) combined poverty-focused microfinance loans with a ten-session participatory curriculum covering topics focused on HIV, IPV, gender norms, and sexuality [32]. A cluster-randomised controlled trial showed positive changes around HIV risk behaviours for young women (under 35 years), including an increase in condom use at last sex with non-cohabiting partners, and greater communication about HIV [32]. Further, past-year sexual and/or physical IPV decreased by more than half [39], and female empowerment indicators improved [40]. A decade later, the IMAGE programme has evolved from a proof of concept to an operational programme, and scaled up to include more than 25,000 households across three South African provinces [41]. To examine the lives of women enrolled in this scaled-up programme, we conducted a cohort study to interview participants right after completion of the ten-session curriculum, and 12 months later, whilst still enrolled in the microfinance programme.

In this cross-sectional paper, we examine the association between income contribution to the household, relationship status, and engagement in risky sexual behaviours among adult women in rural South Africa a year after completing a gender training curriculum and receiving microfinance loans. We hypothesise that relationship status has a significant association with the type of risky sexual behaviours that women engage in, and that increased financial independence may strengthen a woman’s position in choosing safer sexual practices.

## Methods

### Study Design and Setting

The original IMAGE cluster-randomised trial conducted in rural South Africa (2001–2005) combined a poverty-focused microfinance loan programme (administered by the Small Enterprise Foundation (SEF)) with a participatory gender training component (named Sisters for Life (SFL)), and has since been scaled-up to a non-research programme across South Africa [32]. A new cohort study was conducted from 2016 to 2018 to explore the effect of the IMAGE programme on women’s lives a decade after the original trial. Data were collected at two time points: just after receiving the intervention (October–December 2016) and 12 months after its completion (October–December 2018).

This paper is a cross-sectional analysis of data from the follow-up round of the cohort study, a year after participants completed the gender training programme and continued to receive microfinance loans. The study was conducted in rural Mahikeng in the North West province of South Africa. This is an area of widespread poverty, with a general unemployment rate of 35.7%, and among the youth (age 15–34) as high as 47.1% [42]. This study setting was chosen to align with the SFL training sessions commencing in the area in 2016–2017. There were approximately 77 SEF loan centres in the area comprising 460 loan groups (4–8 groups per centre) with a total of 2399 recipients (approximately 5 per loan group) [41].

### Participant Recruitment and Data Collection

To be eligible to participate in the study, women had to be: (i) over 18 years old and (ii) reside in the study site community, (iii) be enrolled at a SEF loan centre and have received microfinance loans for a year or more, and (iv) have recently attended the SFL training sessions. Women were excluded if they were unwilling to participate in the follow-up interview or did not provide written consent. The recruitment of participants took place after loan meetings where SFL sessions were also held. Research supervisors randomly selected 10–20 women from each centre by drawing names from an opaque bag. These women were then explained the interview and consent process and invited to participate. The same women were followed up 12 months later and interviewed again using the same instrument, plus additional questions on HIV perceptions and testing.

Of the 860 women enrolled at baseline, data could be obtained from 626 (73%) in the follow-up round. Data were collected from participants through structured, interviewer-led, tablet-based questionnaires translated into the local language, SeTswana, and back-translated into English to ensure accuracy. Participants were given a unique identification number to maintain confidentiality. Interviews were conducted in a private location by specially trained female interviewers and participants received reimbursement for their time with R50 (~ 3 USD) mobile phone airtime. Ethical approval for the study data collection and analysis was granted by the London School of Hygiene and Tropical Medicine Research Ethics Committee and the Human Research Ethics Committee (Medical) at the University of Witwatersrand, Johannesburg.

### Data Management

Questionnaire modules included: women’s demographic information, relationships, household decision-making, sexual behaviours, HIV testing and risk perceptions, IPV experience, employment, and economic empowerment. Questions about physical, sexual, and emotional IPV were derived from the WHO Multi-country Study on Women’s Health and Domestic Violence survey [43], while economic abuse and controlling partner behaviour questions were adapted from the What Works violence prevention programme in South Africa [44]. Table 1 lists the relevant questionnaire questions regarding participants’ relationship status, household income contribution, and risky sexual behaviours.

**Table 1:** List of questions asked in the IMAGE study about household income contribution, relationship status and risky sexual behaviours

Measure	Question item	Coding
*Outcomes*		
Risky sexual behaviours	Inconsistent condom use: How often have you used condoms when having sex in the last 12 months? Would you say you used them always, often, or sometimes?	Coded ‘1’ if a participant responded “sometimes” or “never” when asked how often they used condoms with any partner in the past 12 months, else “0”, among those who had sex in last 12 months.
	Multiple sexual partners: How many main, secondary, or other partners have you had sex with in the last 12 months?	Coded “1” if a participant responded “two or more” when asked how many sexual partners they had in last 12 months, else “0”.
	Transactional sex: In the past 12 months, have you started or stayed in a main relationship with a partner so that you could receive any of the following: basic household-related costs, somewhere to stay, luxuries for you, cash or money for you, support or money for your household or children?	Coded “1” if participants indicated that they engaged in sex with a main or secondary partner in exchange for material goods in the last 12 months, else “0”.
	In the past 12 months, please think about any man you had sex with just once or any casual partner or secondary partner. Have you entered into a sexual relationship with anyone in order to get things that are important to you, or for help with your expenses, or for money?	
*Exposures*		
Household income contribution	How much of the total monthly household income do you personally earn, based on your work, grants you receive and any other money you generate?	Answers were recorded as ‘none’, ‘half or less’, ‘most of it’, or ‘all of it’.
Relationship status	Are you married or currently living with a man as if married?	Condensed into three levels coded under ‘relationship status’: married or living as married, divorced or separated or widowed, and never married.
	Were you ever married or lived with a man as if married but not now (divorced/widowed)?	
	Do you currently have a main partner but are not living together with him?	
	Do you currently have one or several casual boyfriends/*makwapeni*/secondary partners?	
	Are you currently not in a relationship but have had a partner in the last 12 months? (This can be any partner: husband, main partner or boyfriends/*makwapen*i/secondary partners in the last year)	
	Are you currently not in any relationship with a man and have not been for the last 12 months or more?	

After initial data cleaning, variables were re-inspected for inconsistent or missing answers and certain continuous and categorical variables were re-categorised based on the literature or conceptual reasoning. This was done to avoid sparsity of data, to balance numbers within categories (e.g. age, relationship status), facilitate analyses, or maintain consistency with previous IMAGE analyses [41, 45]. Most of the variables had either no or less than 5% of total missing data. Where there were more than 5% missing in a main outcome variable, as in inconsistent condom use, only those with complete data were included in the analyses.

### Study Measures

#### Outcome and Exposure Variables

We constructed three risky sexual behaviour binary outcome measures: inconsistent condom use, multiple sexual partners, and transactional sex [7, 46]. The two exposure variables were women’s household income contribution and relationship status. A women’s income as a percentage of the household income was determined by asking how much of the total household income she personally earns, whether from work, grants or any other money generated [45] (see Table 1 for details on the construction of variables).

#### Covariates

Age and SES were selected as a priori covariates due to their strong probable association with the exposures and outcomes, as well as prior knowledge derived from literature and similar studies [41, 45]. Age was categorised into five groups (22–34, 35–44, 45–54, 55–64, 65–84 years) to identify age trends in the data, due to the large age range of participants. Household SES was calculated using variables reflecting standard of living, such as ownership of durable assets (e.g. televisions, washing machines), and infrastructure and housing facilities (e.g., sanitation, electricity). Principle component analysis was used to derive an SES index and categorise household SES level into three categories (high, middle or low) [41, 47].

Potential confounders to specific exposure-outcome relationships include: education level, female household headship, number of children under the age of 18 living at home, sexual behaviour exposure measures, age of first sex, any lifetime and past year IPV (constructed as composite variables), and a binary variable created for whether participants underwent an HIV test in past 12 months or not. Women’s relationship power was measured using the Sexual Relationship Power Scale (SRPS, 8 items, Cronbach’s α = 0.70) as previously demonstrated in studies in South Africa [5, 18, 45]. Items were assessed on a 3-point Likert scale from 0 to 24 and each measure categorised into a cut-off level at 50% to create a binary variable of ‘high’ and ‘low’ relationship power for women.

### Statistical Analysis

All statistical analyses were performed using Stata 16.1 (StataCorp, Texas, USA). Descriptive statistics of sociodemographic, sexual behaviour, and IPV factors were performed for the total study population and by age group. We used logistic regression to explore the unadjusted associations between the exposure variables and outcomes (Model 1). Separate multivariable logistic regression models were then constructed for relationship status and household income contribution exposures, to determine their association with each of the risky sexual behaviour outcomes, respectively. First, we adjusted for the a priori variables, age and SES (Model 2), then we further adjusted for additional potential confounders (Model 3). Unadjusted and adjusted ORs, 95% CIs, and p-values were calculated to assess the statistical strength of evidence for associations. Likelihood ratio tests (LRTs) were conducted as general tests for association between the categorical exposures and outcomes.

## Results

### Characteristics of Study Participants

The socio-demographic characteristics of the 626 participants are displayed by age group in Table 2. Participant age varied between 22 and 84 years, and the cohort median age was 51 years. Approximately half of the women (n = 311) were currently married or living as married, 213 (34.1%) were divorced, separated, or widowed, and 101 (16.2%) were single and had never been married. Being divorced, separated, or widowed increased from the youngest to the oldest age category (8.4% to 61.9%), while never being married decreased (47% to 2.4%). More than half of the women (54.1%) did not contribute any income to their households in the past 12 months, 20.1% contributed most of the income, and only 13% all of it. The oldest group had the highest proportion (36.5%) of women with no personal earnings. The women earned money from a diverse range of sources, with the largest proportion earning by providing goods or services in the informal market, followed by government grants or pensions, and formal or part-time employment.

**Table 2:** Distribution of socio-demographic characteristics, IPV and risky sexual behaviours in the study population by age group (n = 626)

Variables	Total sample	Age group (years)					
		18–34	35–44	45–54	55–64	65 +	χ2
	n (%)	n (%)	n (%)	n (%)	n (%)	n (%)	(p-value)
Total participants	626	83 (13.3)	123 (19.7)	167 (26.7)	168 (26.8)	85 (13.6)	
*Socio-demographic characteristics*							
Relationship status	625						
Currently married/living as married	311 (49.7)	37 (44.6)	65 (52.8)	99 (59.3)	80 (47.6)	30 (35.7)	
Divorced/Separated/Widowed	213 (34.1)	7 (8.4)	27 (22.0)	48 (28.7)	79 (47.0)	52 (61.9)	136.12
Never married	101 (16.2)	39 (47.0)	31 (25.2)	20 (12.0)	9 (5.4)	2 (2.4)	(< 0.001)
Education level	626						
Primary school or lower	234 (37.4)	14 (16.9)	29 (23.6)	55 (32.9)	81 (48.2)	55 (64.7)	
Secondary school (any grade)	366 (58.5)	65 (78.3)	88 (71.5)	104 (62.3)	84 (50.0)	25 (29.4)	
Further education	13 (2.1)	3 (3.6)	4 (3.3)	4 (2.4)	1 (0.6)	1 (1.2)	70.61
Other	13 (2.1)	1 (1.2)	2 (1.6)	4 (2.4)	2 (1.2)	4 (4.7)	(< 0.001)
Household SES	626						
Low	209 (33.4)	32 (38.6)	46 (37.4)	51 (30.5)	50 (29.8)	30 (35.3)	
Middle	209 (33.4)	33 (39.8)	43 (35.0)	60 (35.9)	49 (29.2)	24 (28.2)	13.28
High	208 (33.2)	18 (21.7)	34 (27.6)	56 (33.5)	69 (41.1)	31 (36.5)	(0.103)
Woman’s household income contribution	626						
Not earning	151 (54.1)	15 (18.1)	28 (22.8)	34 (20.4)	43 (25.6)	31 (36.5)	
Half or less	268 (42.8)	45 (54.2)	57 (46.3)	77 (46.1)	64 (38.1)	25 (29.4)	
Most	126 (20.1)	13 (15.7)	21 (17.1)	32 (19.2)	40 (23.8)	20 (23.5)	19.47
All	81 (13.0)	10 (12.1)	17 (13.8)	24 (14.4)	21 (15.5)	9 (10.6)	(0.078)
Female household head	626						
Yes	328 (52.4)	44 (53.0)	60 (48.8)	75 (44.9)	97 (57.7)	52 (61.2)	8.96 (0.062)
Number of children < 18 living at home	586						
None	84 (14.3)	8 (10.3)	3 (2.5)	30 (18.3)	25 (16.2)	18 (25.4)	
1– 2	297 (50.7)	37 (47.4)	52 (43.7)	85 (51.8)	84 (54.6)	39 (54.9)	43.37
3 or more	205 (35.0)	33 (42.3)	64 (53.8)	49 (29.9)	45 (29.2)	14 (19.7)	(< 0.001)
*Sexual behaviour and IPV*							
Age of first sex	605						
Under 15	35 (5.8)	4 (4.9)	9 (7.5)	8 (4.9)	8 (5.0)	6 (7.5)	
15–17	203 (33.5)	31 (37.8)	50 (41.7)	55 (33.7)	46 (28.8)	21 (26.3)	10.00
18 or older	367 (60.7)	47 (57.3)	61 (50.8)	100 (61.4)	106 (66.2)	53 (66.2)	(0.265)
Number of sexual partners in last 12 months	626						
None	189 (30.2)	8 (9.6)	18 (14.6)	38 (22.8)	74 (44.1)	51 (60.0)	98.36 (< 0.001)
1	409 (65.3)	65 (78.3)	98 (79.7)	126 (75.4)	89 (53.0)	31 (36.5)	
2 or more	28 (4.5)	10 (12.1)	7 (5.7)	3 (1.8)	5 (3.0)	3 (3.5)	
Condom use in last 12 months	409						
Always	121 (29.6)	21 (29.6)	28 (29.5)	43 (34.7)	26 (28.9)	3 (10.3)	
Often	14 (3.4)	7 (9.9)	2 (2.1)	2 (1.6)	2 (2.2)	1 (3.45)	
Sometimes	73 (17.8)	16 (22.5)	23 (24.2)	23 (18.5)	9 (10.0)	2 (6.9)	33.25
Never	201 (49.1)	27 (38.0)	42 (44.2)	56 (45.2)	53 (58.9)	23 (79.3)	(0.001)
Transactional sex in last 12 months	626						
Yes	41 (6.55)	9 (10.8)	10 (8.1)	9 (5.4)	11 (6.5)	2 (2.3)	5.81 (0.213)
Sexual Relationship Power Scale	448						
Low power	204 (45.5)	33 (44.6)	44 (41.5)	58 (45.3)	44 (44.9)	25 (59.5)	4.01
High power	244 (54.5)	41 (55.4)	62 (58.5)	70 (54.7)	54 (55.1)	17 (40.5)	(0.399)
IPV measures (past 12 months)	626						
Physical violence	31 (4.9)	6 (7.2)	7 (5.7)	8 (4.8)	6 (3.6)	4 (4.7)	1.76 (0.780)
Sexual violence	17 (2.7)	4 (4.8)	3 (2.4)	5 (3.0)	4 (2.4)	1 (1.2)	2.31 (0.679)
Physical and/or sexual violence	38 (6.1)	8 (9.6)	8 (6.5)	9 (5.4)	9 (5.4)	4 (4.7)	2.46 (0.652)
Emotional abuse	97 (15.5)	28 (33.7)	25 (20.3)	26 (15.6)	11 (6.5)	7 (8.24)	36.97 (< 0.001)
Economic abuse	57 (9.1)	15 (18.1)	13 (10.6)	15 (9.0)	9 (5.4)	5 (5.9)	12.30 (0.015)
Any IPV	126 (20.1)	34 (41.0)	32 (26.0)	33 (19.7)	17 (10.1)	10 (11.8)	39.25 (< 0.001)
IPV measures (lifetime)	626						
Physical violence	134 (20.6)	24 (28.9)	20 (16.3)	31 (18.6)	39 (23.2)	20 (23.5)	6.07 (0.194)
Sexual violence	57 (9.11)	11 (13.2)	11 (8.9)	14 (8.38)	18 (10.7)	3 (3.5)	5.55 (0.235)
Physical and/or sexual violence	145 (23.2)	27 (32.5)	21 (17.1)	33 (19.8)	44 (26.2)	20 (23.5)	8.61 (0.072)
Economic abuse	136 (21.7)	27 (32.5)	25 (20.3)	35 (21.0)	35 (20.8)	14 (16.5)	7.36 (0.118)
Any IPV	225 (35.9)	45 (54.2)	46 (37.4)	53 (31.7)	55 (32.7)	26 (30.6)	15.24 (0.004)

Sixty-five percent of women had one sexual partner in the past year, 4.5% had two or more, and 30.2% were not sexually active. Only 9.6% of women aged 22–34 years had no sexual partners, compared to 60% of women over 65, a trend seen with increasing age. The youngest group reported the highest frequency of past year multiple partnerships (12.1%). Almost half (49.1%) of the 409 sexually active women reported never using a condom in the past 12 months. Forty-one women (6.6%) engaged in transactional sex during the past year. A reported 20.1% of women experienced any act of IPV at least once in the past 12 months, with the most prominent type being emotional abuse (15.5%). The youngest women experienced the highest levels of abuse, with 41% experiencing some form of past year IPV.

### Individual Factors Associated with Risky Sexual Behaviours

There was very strong evidence of a crude association between relationship status and past year inconsistent condom use (χ2 = 29.40, p < 0.001); women married or living as married had more than three times higher odds of not using a condom consistently, compared with never married women (OR = 3.58, 95%CI 2.10–6.22). There was strong evidence of age only being associated with multiple sexual partnerships (χ2 = 13.76, p = 0.008). With the over-65 s as reference, only the 55–64 years group had lower odds of having multiple partners (OR = 0.88, 95%CI 1.26–13.41). For details, see Table 3.

**Table 3:** Unadjusted association between the exposures, covariates, and past year inconsistent condom use, multiple sexual partners, and transactional sex as outcomes

Variables	N (%)	Risky sexual behaviour outcomes (past 12 months)								
		Inconsistent condom use			Multiple sexual partners			Transactional sex		
		OR	95%CI	χ2 (p-value)	OR	95%CI	χ2 (p-value)	OR	95%CI	χ2 (p-value)
*Exposures*:										
Relationship status	625									
Never married	311 (49.7)	1		29.40 (< 0.001)	1		1.77 (0.412)	1		3.86 (0.145)
Divorced/separated/widowed	213 (34.1)	1.26	[0.64–2.49]		0.85	[0.38–1.85]		0.40	[0.16–1.03]	
Married/living as married	101 (16.2)	3.58	[2.10–6.22]		0.62	[0.29–1.34]		0.69	[0.31–1.52]	
Woman’s household income contribution	626									
None	151 (54.1)	1		1.95 (0.583)	1		14.86 (0.002)	1		11.09 (0.011)
Half or less	268 (42.8)	0.96	[0.56–1.63]		9.74	[2.24–42.42]		0.44	[0.21–0.90]	
Most	126 (20.1)	0.67	[0.34–1.31]		6.42	[1.35–30.59]		0.18	[0.05–0.64]	
All	81 (13.0)	0.77	[0.37–1.63]		10.49	[2.13–51.61]		0.49	[0.17–1.37]	
*Covariates*:										
Age	626									
65 +	85 (13.6)	1		6.99 (0.136)	1		13.76 (0.008)	1		5.81 (0.214)
55–64	168 (26.8)	0.35	[0.11–1.14]		0.88	[1.26–13.41]		2.91	[0.62–13.55]	
45–54	167 (26.7)	0.28	[0.10–0.88]		2.30	[0.61–6.51]		2.36	[0.49–11.27]	
35–44	123 (19.7)	0.35	[0.11–1.12]		1.99	[0.74–7.10]		3.67	[0.77–17.47]	
18–34	83 (13.3)	0.25	[0.07–0.82]		4.11	[0.25–3.10]		5.05	[1.02–24.80]	
SES	626									
Low	209 (33.4)	1		0.98 (0.613)	1		1.19 (0.552)	1		1.50 (0.473)
Middle	209 (33.4)	1.14	[0.68–1.90]		0.87	[0.42–1.80]		0.84	[0.36–1.92]	
High	208 (33.2)	1.29	[0.77–2.16]		1.27	[0.65–2.48]		1.34	[0.63–2.84]	
Education level	626									
Primary or below	234 (37.4)	1		3.89 (0.274)	1		17.69 (0.001)	1		3.72 (0.294)
Secondary school (any grade)	366 (58.5)	1.03	[0.67–1.60]		1.45	[0.77–2.73]		1.59	[0.79–3.19]	
Further education	13 (2.1)	0.71	[0.21–2.38]		9.13	[2.53–32.86]		-	-	
HIV test (past 12 months)	626									
No	155 (24.8)	1		0.37 (0.545)	1		4.14 (0.042)	1		2.41 (0.121)
Yes	471 (75.2)	0.85	[0.51–1.42]		2.29	[1.01–5.20]		1.99	[0.82–4.84]	
Any IPV (past 12 months)	626									
No	500 (79.9)	1		1.25 (0.263)	1		3.64 (0.056)	1		2.28 (0.131)
Yes	126 (20.1)	0.77	[0.48–1.22]		1.82	[0.97–3.40]		1.71	[0.84–3.46]	
Any IPV (lifetime)	626									
No	401 (64.1)	1		0.58 (0.448)	1		0.00 (0.988)	1		1.21 (0.272)
Yes	225 (35.9)	0.85	[0.56–1.30]		0.99	[0.55–1.79]		1.43	[0.75–2.71]	
Relationship power	448									
High	204 (45.5)	1		0.33 (0.568)	1		0.13 (0.715)	1		0.09 (0.770)
Low	244 (54.5)	1.13	[0.74–1.74]		0.89	[0.47–1.69]		0.90	[0.46–1.78]	
Age of first sex	605									
Under 15	35 (5.8)	1		0.93 (0.629)	1		1.63 (0.442)	1		0.22 (0.894)
15–18	203 (33.5)	0.87	[0.34–2.23]		3.51	[0.45–27.42]		0.73	[0.20–2.71]	
Over 18	367 (60.7)	0.72	[0.29–1.81]		3.14	[0.41–23.8]		0.78	[0.22–2.73]	
Household head gender	626									
Male	298 (47.6)	1		40.99 (< 0.001)	1		0.86 (0.353)	1		0.03 (0.867)
Female	328 (52.4)	0.25	[0.16–0.40]		1.31	[0.74–2.32]		1.05	[0.56–1.99]	

Women’s household income contribution showed strong evidence of a crude association with multiple sexual partners and transactional sex (χ2 = 14.86, p = 0.002 and χ2 = 11.09, p = 0.011). Those contributing no income had the lowest odds of having multiple partners, but the highest odds of engaging in transactional sex. Those contributing most of the income had the lowest odds of transactional sex (OR = 0.18, 95%CI 0.05–0.64), while those who contributed all of the income had ten-fold higher odds of having multiple partners (OR = 10.49, 95%CI 2.13–51.61) compared to those with no contribution. There was some evidence that those who experienced any past year IPV had higher odds of multiple partnerships (χ2 = 3.64, p = 0.056). There was very strong evidence demonstrating that women in female-headed households had much lower odds of inconsistent condom use compared to those headed by males (OR = 0.25, 95%CI 0.16–0.40, p < 0.001). Finally, education showed a strong statistically significant association only with multiple sexual partnerships (χ2 = 17.69, p < 0.001), and women with post-secondary school education had more than nine times higher odds of multiple partnerships than those with primary school or lower, albeit with a wide confidence interval (OR = 9.13, 95%CI 2.53–32.86).

### Association Between Women’s Household Income Contribution and Risky Sexual Behaviours

We also conducted multivariable analysis to test the association between women’s household income contribution and risky sexual behaviours (see Table 4). In the adjusted model, income contribution showed strong evidence of an association with multiple sexual partners (χ2 = 15.90, p = 0.001). Women who contributed any proportion of income, whether half or less (aOR = 8.55, 95%CI 1.98–36.86) or all (aOR = 8.94, 95%CI 1.87–42.76), had significantly higher odds of having multiple sexual partners versus women contributing no income to their households. Conversely, in the full adjustment, those who contributed most of the income had the lowest odds of engaging in transactional sex (aOR = 0.18, 95%CI 0.05–0.63, p = 0.011) compared to those who contributed nothing. There was no evidence of an association between household income and inconsistent condom use (χ2 = 1.34, p = 0.721).

**Table 4:** Multivariable logistic regression models for the association between women’s  share of household income contribution and past year inconsistent condom use, multiple sexual partners, and transactional sex

Sexual risk behaviour	Model 1: Unadjusted			Model 2: Partially adjusted ^a^			Model 3: Fully adjusted ^b^		
	OR	95%CI	LRT χ2 (p-value)	aOR	95%CI	LRT χ2 (p-value)	aOR	95%CI	LRT χ2 (p-value)
*Inconsistent condom use*			(n = 409)			(n = 409)			(n = 402)
None	1		1.92 (0.589)	1		1.85 (0.604)	1		1.34 (0.721)
Half or less	0.96	[0.56–1.63]		1	[0.58–1.72]		0.89	[0.50–1.60]	
Most of it	0.67	[0.35–1.31]		0.7	[0.36–1.37]		0.71	[0.34–1.48]	
All of it	0.77	[0.37–1.62]		0.78	[0.37–1.67]		1.12	[0.50–2.53]	
*Multiple sexual partners*			(n = 626)			(n = 626)			(n = 612)
None	1		19.42 (< 0.001)	1		16.66 (0.001)	1		15.9 (0.001)
Half or less	9.74	[2.30–41.32]		8.63	[2.02–36.83]		8.55	[1.98–36.86]	
Most of it	6.42	[1.38–29.88]		6.44	[1.37–30.17]		5.14	[1.07–24.68]	
All of it	10.49	[2.24–49.15]		9.86	[2.09–46.59]		8.94	[1.87–42.76]	
*Transactional sex*			(n = 626)			(n = 626)			(n = 599)
None	1		10.91 (0.012)	1		12.39 (0.006)	1		11.12 (0.011)
Half or less	0.44	[0.21–0.90]		0.37	[0.18–0.78]		0.39	[0.19–0.83]	
Most of it	0.18	[0.05–0.63]		0.17	[0.05–0.59]		0.18	[0.05–0.63]	
All of it	0.49	[0.17–1.36]		0.43	[0.15–1.23]		0.44	[0.15–1.26]	

a – Model 1 adjusted for forced variables (age and SES)

b – Model 2 adjusted for education level, relationship status, any IPV experienced in the last 12 months, household head gender

### Association Between Relationship Status and Risky Sexual Behaviours

We conducted a multivariable analysis for the association between relationship status and inconsistent condom use, multiple sexual partners, and transactional sex (see Table 5). Those who were married or living as married had the highest odds of inconsistent condom use compared to those previously or never married in the fully adjusted model (χ2 = 27.33, p < 0.001). For multiple sexual partnerships after full adjustment, there was some evidence that divorced, separated, or widowed women had more than twice higher odds of having multiple partners compared to currently married women (aOR = 2.29, 95%CI 1.10–4.75). For transactional sex, there was weak evidence of an association with relationship status in the unadjusted model (χ2 = 3.89, p = 0.143). In the fully adjusted model, there was no evidence of an association (χ2 = 0.37, p = 0.834).

**Table 5:** Multivariable logistic regression models for the association between relationship status and past year inconsistent condom use, multiple sexual partners, and transactional sex

Sexual risk behaviour	Model 1: Unadjusted			Model 2: Partially adjusted ^a^			Model 3: Fully adjusted ^b^		
	OR	95%CI	LRT χ2 (p-value)	aOR	95%CI	LRT χ2 (p-value)	aOR	95%CI	LRT χ2 (p-value)
*Inconsistent condom use*			(n = 409)			(n = 409)			(n = 409)
Married/living as married	1		28.75 (< 0.001)	1		28.44 (< 0.001)	1		27.33 (< 0.001)
Divorced/separated/widowed	0.35	[0.20–0.62]		0.32	[0.17–0.58]		0.32	[0.17–0.59]	
Never married	0.28	[0.16–0.47]		0.27	[0.15–0.49]		0.27	[0.15–0.48]	
*Multiple sexual partners*			(n = 625)			(n = 625)			(n = 612)
Married/living as married	1		1.76 (0.416)	1		4.63 (0.099)	1		5.12 (0.077)
Divorced/separated/widowed	1.36	[0.72–2.56]		2.11	[1.06–4.19]		2.29	[1.10–4.75]	
Never married	1.61	[0.75–3.44]		1.07	[0.47–2.43]		1.11	[0.47–2.65]	
*Transactional sex*			(n = 625)			(n = 625)			(n = 599)
Married/living as married	1		3.89 (0.143)	1		1.23 (0.540)	1		0.37 (0.831)
Divorced/separated/widowed	0.58	[0.26–1.28]		0.68	[0.30–1.57]		0.8	[0.34–1.92]	
Never married	1.44	[0.66–3.16]		1.18	[0.51–2.75]		1.11	[0.46–2.64]	

a – Model 1 adjusted for forced variables (age and SES)

b – Model 2 adjusted for education level, women’s household income contribution, any IPV experienced in the last 12 months

## Discussion

In this paper, we explore the association between women’s income contribution and the status of their relationships on risky sexual behaviours in the context of a microfinance-plus programme in rural South Africa. Women’s income contribution was strongly associated with having multiple sexual partners and engagement in transactional sex, but showed no association with inconsistent condom use. We also show a strong association between relationship status and inconsistent condom use, with married women having the highest odds of unprotected sex in the past year, and never married women the lowest. There was some evidence to suggest that relationship status was associated with having multiple sexual partners, as women who were divorced, separated, or widowed were twice as likely to have had two or more sexual partners in the past year, compared to the other groups.

Many HIV prevention intervention programmes focus on young women of reproductive age, and rightly so, but they often exclude older women whose HIV risk remains substantial [48]. Women over 50 years are more likely to be at late-stage HIV infection by the time of diagnosis, as they often mistake their symptoms for natural aging and are less likely to discuss sexual health with their healthcare providers. This means they are often only diagnosed when treatment for other diseases fail [49]. In this study, where the median participant age was 51 years old, the results show strong evidence that married women and older women were significantly more likely to use condoms inconsistently. Other studies from South Africa showed similar findings [14, 50]. A study in the same region as the current study found that only 16.2% of married or cohabitating women consistently used condoms [51]. Being married is not necessarily a protective factor for HIV infection, as these women may be unaware of their partner’s sexual history or concurrent partnerships [52, 53]. The main reasons for inconsistent condom use in long-term partnered women were low sexual decision-making power, fear of relationship instability, and fear of infidelity accusations [51]. The widespread patriarchal culture in South Africa and the pervasive social norms around the general acceptance of husbands’ infidelity mean that women often have little or no agency in sexual decision making within a marriage. Suggesting condom use or declining unprotected sex is seen as a challenge to a husband’s authority or a sign of unfaithfulness, and may be met with physical violence, emotional abuse, or forced sex [50, 53, 54].

Our findings of increased odds of multiple partnerships among those who contribute more income to their households is unexpected, but similar to the findings of a study in Botswana, where women earning the same or more than their partner was significantly associated with having more sexual partnerships [55]. Higher income contribution and related financial independence may be associated with an increase in power, confidence, and sexual agency, leading to an increased number of sexual partnerships. This agency and independence may also make it easier for women to leave relationships, leading to more partnerships over a period of time [56, 57]. Women who contributed no income to the household and were entirely financially dependent on their partners or families had the lowest odds of having multiple sexual partners, but the highest odds of engaging in transactional sex. These findings partially contradict other studies, where transactional sex is usually associated with other risky sexual behaviours, including multiple sexual partnerships [58, 59]. It suggests that a lack of income pushes women towards engaging in transactional sex, but potentially with partners with whom they have longer term relationships. Women who engage in transactional sex are more vulnerable to HIV infection due to the power imbalance in these relationships, especially when there is a large age-difference, since they often have little to no say in the conditions under which sexual encounters take place [24, 27]. Men who engage in transactional sex are generally older, have multiple partners, use condoms inconsistently, and have an increased likelihood of being HIV infected compared to the general male population, placing their female partners at a largely increased risk [26, 60]. While women’s engagement in transactional sex is often motivated by basic needs or food insufficiency [61–63], it may also be driven by the pressure to obtain certain fashionable or luxury items to improve social standing, especially among younger women and those who could not afford it otherwise [24, 25, 64]. For women in the ‘sex for basic needs’ category, having some form of financial income, through cash transfer interventions for example, has been shown to improve their agency in rejecting certain types of unsafe or unwanted sexual relationships they might have otherwise turned to due to limited resources [65]. Our findings suggest that women may ‘transition’ from engaging in transactional sex (being in a position of lower power once agreeing to sex), to multiple sexual partnerships (where they are in a position of greater agency) as their income and financial autonomy increases. Women’s control of their own money has been found significantly associated with HIV-related negotiation, which highlights the role of financial autonomy in giving women the choice to protect themselves from HIV infection [66].

By providing women with a source of independent income and gender training, microfinance-plus interventions are effective by addressing the broader social determinants of health [35, 39, 40]. Thus, considering the broader implications for women’s health and empowerment, focusing more on structural drivers (e.g. economic insecurity, harmful gender and social norms) rather than individual behaviours might be more beneficial in addressing the inequalities that place women in unsafe sexual situations.

Certain limitations of this study must be noted. Due to its cross-sectional nature, it is not possible to infer causality in any of the associations. It also means that the temporality of the associations could not be established, since it was not possible to determine exactly when the exposure or outcome took place within the past year. These considerations, combined with previous literature demonstrating the bidirectionality of many relationships, meant that statistical associations were only described as such, and no attempts were made to infer temporality. Although attempts were made to include participants of diverse ages and marital statuses, all of the participants were women from rural, low-income communities and active participants of the microfinance-plus scheme. Therefore, the results of this study may not be completely generalisable to other contexts. The analyses had to be simplified and performed with the data available, and therefore residual confounding remains a possibility. For example, substance abuse was identified as an important risky sexual behaviour but was not covered in the questionnaire. Attempts were made to minimise selection bias by manually randomising the participants at the loan centre selection process. However, the exposures and outcomes were self-reported, so recall bias is likely and social desirability bias may have caused women to underreport negatively viewed sexual behaviours, a phenomenon often seen in condom-use self-reporting [67]. An important element not included in this study is the women’s male partner perspectives. Further qualitative studies that include focus group discussions with individuals and couples would provide valuable context on men’s attitudes towards the microfinance-plus programme, and perspectives on HIV risk.

## Conclusion

This study reinforces the complexity and multi-directionality of the relationships between women’s household income contribution, relationship status and sexual risk behaviour. Older women are often neglected in sexual risk behaviour research and interventions, though their HIV risk should not be ignored. Therefore, interventions (including the complementary programmes around gender training provided alongside economic interventions) should be responsive to the evolving sexual health vulnerabilities of women across their entire life course. Economic and social empowerment interventions have proven effective in HIV prevention efforts through addressing structural drivers of HIV risk. However, these programmes need to be combined with activities that include male engagement and address unequal gender norms. The recent COVID-19 pandemic has exacerbated many inequalities, including gendered poverty and related relationship power imbalances. This reaffirms the pressing need for evidence-based programming that focuses on skills building, gender training and income generation relevant to both younger and older women such contexts.

## Data Availability

The datasets generated and analysed during this current study are not publicly available for 10 years as they contain participant identification numbers. A de-identified dataset will be made available after this time in the London School of Hygiene and Tropical Medicine data repository.

## Code Availability

N/A.
